# 

**Published:** 1895-12

**Authors:** 


THE MISSOURI VALLEY VETERINARY MEDICAL ASSOCIATION will hold their regular quarterly meeting at Kansas City, on December 11, 1895. Clinical demonstration and a full program await those who may be favored by attendance.

THE INDIANA VETERINARY ASSOCIATION OF GRADUATES will hold a meeting at Muncie on December 10th and 11th. An interesting program and an equine-flesh dinner are among the attractions.

### PERSONAL.

Dr. J. W. Sallade, of Pottsville, Pa., recently met with painful injuries through a runaway accident, but is now slowly convalescing.

Mr. A. W. Bitting, of Purdue University, has obtained a veterinary degree at the Veterinary Department at Ames, Iowa.

Dr. J. McBirney, formerly of Charles City, Iowa, has recently been assigned to duty at Philadelphia as an assistant meat-inspector.

Dr. A. R. Wake, late house-surgeon of the Veterinary Department at Ames, Iowa, has received an appointment as meat-inspector under the Bureau of Animal Industry, and has been assigned to duty at Chicago.

F. T. Eisenman, M.D., V.S., of Louisville, Ky., fills the post of State veterinarian for the blue-grass commonwealth.

Dr. D. LeMay, of Ft. Grant, Arizona, has been out of service for three weeks, owing to serious injuries received, the result of a fall.

Dr. S. B. Nelson, of Spokane, Wash., has received the appointment as veterinarian to the experiment station, and also that of State veterinarian. Dr. Nelson is a graduate of the Veterinary Department of the Iowa Agricultural College, a member of the United States Veterinary Medical Association, and his appointment is well merited.

